# The Correlation of the Tinnitus Handicap Inventory with Depression and Anxiety in Veterans with Tinnitus

**DOI:** 10.1155/2015/689375

**Published:** 2015-11-30

**Authors:** Jinwei Hu, Jane Xu, Matthew Streelman, Helen Xu, O'neil Guthrie

**Affiliations:** ^1^Department of Otolaryngology and Head & Neck Surgery, Loma Linda University Medical Center, Loma Linda, CA, USA; ^2^Loma Linda Veterans Affairs Medical Center, Loma Linda, CA, USA; ^3^Loma Linda University Medical School, Loma Linda, CA, USA; ^4^Cell & Molecular Pathology Laboratory, Department of Communication Sciences and Disorders, Northern Arizona University, Flagstaff, AZ, USA

## Abstract

*Objective*. The mechanisms of tinnitus are known to alter neuronal circuits in the brainstem and cortex, which are common to several comorbid conditions. This study examines the relationship between tinnitus and anxiety/depression.* Subjects and Methods*. Ninety-one male veterans with subjective tinnitus were enrolled in a Veterans Affairs Tinnitus Clinic. The Tinnitus Handicap Inventory (THI) was used to assess tinnitus severity. ICD-9 codes for anxiety/depression were used to determine their prevalence. Pure tone averages (PTA) were used to assess hearing status.* Results*. Descriptive analyses revealed that 79.1% of the 91 tinnitus sufferers had a diagnosis of anxiety, 59.3% had depression, and 58.2% suffered from both anxiety/depression. Patients with anxiety had elevated total THI scores as compared to patients without anxiety (*p* < 0.05). Patients with anxiety or depression had significantly increased Functional and Emotional THI scores, but not Catastrophic THI score. Significant positive correlations were illustrated between the degree of tinnitus and anxiety/depression (*p* < 0.05). There were no differences in PTA among groups.* Conclusions*. A majority of patients with tinnitus exhibited anxiety and depression. These patients suffered more severe tinnitus than did patients without anxiety and depression. The data support the need for multidisciplinary intervention of veterans with tinnitus.

## 1. Introduction

Subjective tinnitus is an acoustic sensation perceived in the absence of an auditory stimulus. The sounds heard by tinnitus sufferers can be a ringing, buzzing, chirping, roaring, and/or a large variety of other types of sounds. The sound may be intermittent or constant and may localize to the right ear, left ear, either ear, or neither ear but instead may be perceived in the head. Epidemiology studies report that 15%–20% of the adult populations experience some form of tinnitus, either temporarily or permanently [1]. Many people can cope with chronic tinnitus, but for 1-2% of the population, it is a severe handicap significantly impairing their quality of life [2]. According to the American Tinnitus Association, fifty million Americans suffer with tinnitus and tinnitus is the most prevalent service connected disability among veterans (exceeding posttraumatic stress disorder and traumatic brain injury). The Department of Veterans Affairs (VA) has reported that tinnitus disability claims have exceeded 840,000 and the cost to compensate veterans for tinnitus is over $1.28 billion annually. A wide variety of therapies exist for tinnitus, but none have been consistent in providing relief. This is due, in part, to the fact that tinnitus patients have not been clustered in such a way that determines the best treatment approach for individual patients.

Tinnitus is believed to be a sign of dysfunctional auditory neurons [3]. For instance, loud noise exposure is the most prevalent and direct cause of tinnitus and loud noise exposure can induce primary neuropathy of the VIIIth craniofacial nerve (primary auditory neurons) whether the hearing loss recovers or not [4]. Additionally, tinnitus is an early warning sign of auditory neoplastic neuromas [5]. Furthermore, patients with normal hearing and tinnitus reveal deafferentation of high threshold VIIIth nerve fibers with normal low and mid threshold fibers, indicating that only a subset of neurons may drive tinnitus [3]. These and other evidences demonstrate that tinnitus is a signal for the presence of dysfunctional auditory neurons. However, not all auditory neuronal dysfunctions lead to tinnitus and therefore tinnitus may represent a specific type of dysfunction among a specific population of neurons.

Tinnitus may start within the VIIIth nerve but the generating loci may shift with time to more central (brainstem and cortex) regions [6]. This suggests that the particular auditory neuronal dysfunction that drives tinnitus will also affect the central nervous system. This is plausible, given that certain lesions to the VIIIth nerve result in the reorganization of tonotopic maps in the cortex and more important for tinnitus, results in an increase in neuronal excitability from the brainstem to the cortex [7]. Recent literature has shown that up to 77% of the tinnitus population may present with psychiatric comorbidities [2, 8, 9]. Among those psychiatric disorders, anxious and depressive symptoms seem to be the most common complications with tinnitus [10], with a lifetime prevalence of depression and anxiety significantly higher in tinnitus patients than in the general population [9].

When considering psychological comorbidities such as anxiety/depression with tinnitus, the disease may be further categorized into compensated versus decompensated tinnitus. In compensated tinnitus, the patient copes well with the tinnitus and there is little or no psychological strain. On the other hand, in decompensated tinnitus, the tinnitus perception is considered uncontrollable and interferes with the patient's quality of life, causing associated emotional and psychological distress [10].

In compensated tinnitus patients, the perception of auditory sounds is normally extinguished in a short time through the “habituation” mechanism: the superior brain (involving the frontal gyri, cingulate gyrus, and parietal cortices) activates thalamic filters to “switch off” the signal, often independently of the resolution of the dysfunction that generated the tinnitus (peripheral auditory nerve dysfunction and neural changes of the central auditory system). This mechanism was initially identified by Dehaene and Changeux as components a “global neuronal workspace” that is activated by normal hearing subjects when required to consciously process task stimuli and make behavioral responses [11]. It has recently been proposed that engagement of the global workspace is essential for the conscious experience of a tinnitus sound [12]. In other words, the perception of tinnitus requires active awareness of the sensation, and shifting attention extinguishes the perception of the sounds. This understanding plays a significant role in medical therapy for tinnitus, as is evidenced in the use of “masking” techniques.

In decompensated tinnitus, there is usually a negative emotional reaction such as fear, anxiety, or tension associated with the perception of the sounds. Psychology literature illustrates that negative appraisals of events and situations produce distorted misinterpretations of the events, leading to memory and attention strategies that recall negative elements in the environment during the event [13, 14]. Based on this model, Fagelson proposed that the individual suffering from tinnitus could develop emotional disorders such as anxiety and depression that were triggered or heightened by an inappropriate interpretation of a sensory event [13]. While Fagelson studied a cohort who also suffered from PTSD (posttraumatic stress disorder) and this disorder was not included in our patient population, the finding of these investigators can only be compared with ours with qualification. In this context, negative emotional reinforcement may interfere with the habituation mechanism by drawing more attention to the perception and prolonging the event. Although current literature is still inconclusive on whether tinnitus leads to anxiety and depression or vice versa, substantial physiological evidence through PET and fMRI studies suggests that negative emotions and continued perception of tinnitus are amplified in a vicious negative feedback loop. The model proposed by Georgiewa et al. [15] and Hazell and Jastreboff [16] describes continued perception of tinnitus as being supported by the amygdala, which is also activated by negative emotions. When tinnitus and negative emotions are present together, there is amplification (via increased neuronal excitability) and chronification (via neural plasticity mechanism) of signals, resulting in persistence of both emotional and tinnitus symptoms.

Because of the strong association of tinnitus with psychiatric disorders and indications that veterans are particularly vulnerable to experiencing tinnitus, anxiety, and depression [17], this study aimed to further evaluate comorbid anxiety and depression associated with tinnitus in a veteran population.

## 2. Subjects and Method

Ninety-one male veterans who reported subjective tinnitus were enrolled in a VAMC Tinnitus Clinic from 2010 to 2013. A retrospective chart review of case history, audiometric thresholds, self-assessment of tinnitus handicap, and ICD-9 codes was conducted on all patients. The Institutional Review Board (IRB) at the VAMC approved all protocols.

Data from the medical records included demographic information, tinnitus case history, audiologic case history, pure-tone thresholds, and information contained in self-assessment inventories. The Tinnitus Handicap Inventory (THI) [18] was used to assess tinnitus severity. The diagnoses of anxiety and depression were established through screening and intake examinations conducted by the Behavioral Medicine Service at the VAMC. ICD-9 codes for anxiety and depression were used to identify patients with these diagnoses and were recorded as categorical data for analysis (e.g., 1 = anxiety, 0 = no anxiety). Pure-tone averages (PTAs) at 500, 1000, and 2000 Hz were used to assess hearing status.

## 3. Statistics

For the purpose of analyzing the effect of anxiety and depression on tinnitus, the patients were divided into five groups: tinnitus with anxiety, tinnitus without anxiety, tinnitus with depression, tinnitus without depression, and tinnitus with both anxiety and depression (see Table 1). SPSS (version 21; IBM Corporation, Armonk, NY) was used for statistical analysis. *p* value was considered significant when *p* < 0.05.

## 4. Results

### 4.1. Demographic Information

Among the 91 male veterans with subjective tinnitus, 72/91 (79.1%) were diagnosed with anxiety; 19/91 (20.9%) were without anxiety; 54/91 (59.3%) were diagnosed with depression; 37/91 (40.6%) were without depression; and 53/91 (58.2%) were diagnosed with both anxiety and depression. Table 1 lists the median age, standard deviations, and age ranges among five groups. There is no significant age difference among the five groups, with the median age being 64.

### 4.2. Hearing Loss and Tinnitus

**Figure 1:**
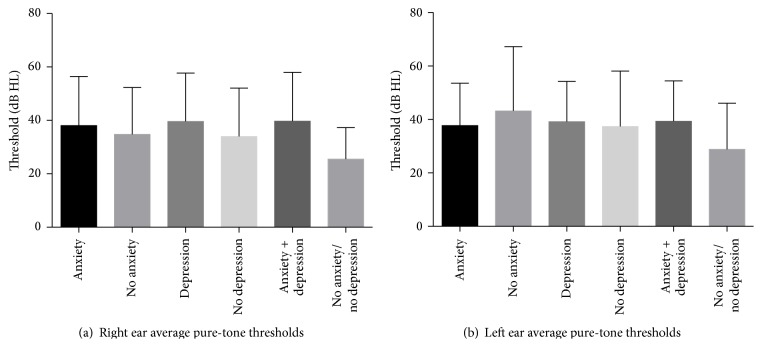
The pure-tone average (500, 1000, and 2000 Hz) of both ears among tinnitus patients with and without anxiety and depression.

The mean PTAs showed no differences among the five groups or between ears (*p* > 0.05). The average hearing loss was around 38 dB HL among all the groups (Figure 1). A bivariate plot of the PTAs and the THI scores was performed and indicated that both the PTAs and THI scores displayed substantial variability, and the comparison of means demonstrated that self-assessed handicap between the groups was independent of auditory thresholds (data no shown).

### 4.3. Anxiety, Depression, and Tinnitus

**Figure 2:**
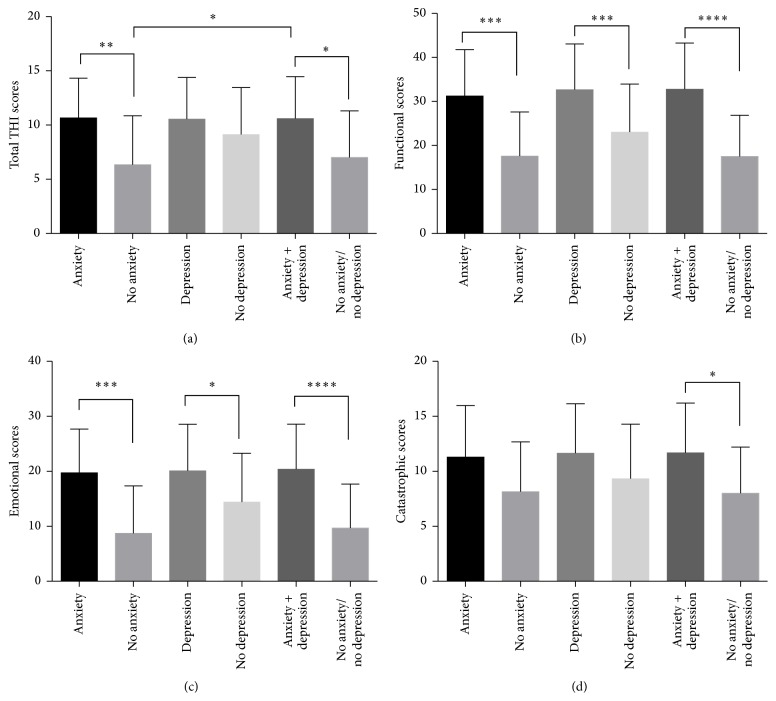
Tinnitus severity presented with total THI (a) and functional (b), emotional, (c) and catastrophic subscales (d) among tinnitus patients with and without anxiety and depression. ^*∗*^ *p* < 0.05; ^*∗∗*^ *p* < 0.01; ^*∗∗∗*^ *p* < 0.001; and ^*∗∗∗∗*^ *p* < 0.0001.

Tinnitus severity was assessed with the THI. The THI is a 25-item self-report questionnaire that has functional, emotional, and catastrophic subscales.  Figure 2(a) shows that patients with anxiety had elevated total THI scores as compared to patients without anxiety (*p* < 0.01). Patients with both anxiety and depression had elevated total THI scores as compared to patients without anxiety (*p* < 0.01). However, there was no significant difference between patients with or without depression (*p* > 0.05).  Figure 2(b) shows that patients with either anxiety or depression had significantly increased functional THI scores (*p* < 0.01). Similar to the functional subscale, patients with either anxiety or depression had significantly increased emotional THI scores (*p* < 0.01) (Figure 2(c)). However, there was no statistical difference among the five groups in the catastrophic THI subscale (*p* > 0.05) (Figure 2(d)). Interestingly, patients with both anxiety and depression do not have worsening subscale scores as compared to patients with either condition (Figure 2). There is no significant difference between patients with neither anxiety nor depression versus patients with no anxiety or no depression only in terms of THI and subscale scores (Figure 2).

### 4.4. Correlations between the Degree of Tinnitus and Anxiety/Depression

Pearson correlation analysis was performed between scores for tinnitus and anxiety or depression. These are reported in Tables 2(a) and 2(b). Functional THI scores had the strongest correlation with both anxiety and depression, followed by emotional scores. The data also demonstrated that anxiety has higher correlation to more severe tinnitus in terms of total THI score than depression.

### 4.5. Association between Tinnitus Characteristics and Comorbid Anxiety/Depression

Tinnitus characteristics such as occurrence (e.g., intermittent and persistent), lateralization (unilateral and bilateral), and years of suffering were evaluated among patients with and without anxiety/depression (see Table 3). The prevalence of anxiety was higher among patients with intermittent tinnitus relative to those who suffer with persistent tinnitus. However, depression was more prevalent among those who suffered with persistent tinnitus relative to intermittent tinnitus. The prevalence of anxiety was higher among patients who suffered with unilateral tinnitus relative to those with bilateral tinnitus. In contrast, the prevalence of depression was higher among patients with bilateral tinnitus compared with patients with unilateral tinnitus. Interestingly, both anxiety and depression tend to be more prevalent after 10 years of suffering with tinnitus. These results suggest that the characteristics of the tinnitus perception may be associated with comorbid conditions.

## 5. Discussion

### 5.1. Association of Tinnitus with Anxiety/Depression

Subjective tinnitus, as opposed to objective tinnitus with a physical stimulation generator within the body, is by far the more common type of tinnitus experienced by patients. Various studies have shown a positive correlation between the severity of tinnitus and quantitative measures of anxiety and depression. Unterrainer et al. showed that comorbid depression was one of the best predictors for perceived severity of tinnitus, greater than duration, pitch, locus of control, or the perception of tinnitus as an illness [19]. According to Zöger et al., increased severity of anxiety and depression is associated with more severe tinnitus [20]. This finding was replicated by Zeman et al. using the THI questionnaire, underscoring the usefulness of the THI as a screening instrument for comorbid depression and anxiety [21]. The same study also found that 15 out of the 25 items of the THI are significantly related to the Beck Depression Inventory (BDI) and explain more variance of BDI than the total THI score, although the authors suggest the THI should still be considered a one-factor instrument in explaining quality of life. Similar to the above studies, we demonstrated positive correlations between the severity of tinnitus and anxiety or depression in a VA population. Furthermore, tinnitus patients presented with anxiety (79.1%) and depression (59.3%) were considerably higher than the civilian population [2, 8, 9, 22]. This finding might suggest that veterans with tinnitus present with a higher prevalence of anxiety and depression than the general population; however more extensive analysis of larger veterans cohorts will likely be needed to clarify such a predilection.

When comparing anxiety against depression, Granjeiro et al. showed that patients with depression had milder tinnitus symptoms while patients with anxiety had more moderate tinnitus symptoms, and patient with both anxiety and depression together had the most severe tinnitus symptoms [22]. The same study also showed that higher anxiety and depression scores correlated with higher tinnitus severity scores [22]. In addition, our study showed that anxiety has higher correlation to more severe tinnitus than depression, consisting with Granjeiro et al.'s study [22]. However, our study showed that patients with both anxiety and depression do not exhibit worsening tinnitus symptoms as compared to patients with anxiety or depression alone, which was different from the above study [22].

Interestingly, Folmer et al. found no correlation between depression and loudness of perceived tinnitus, but there was a positive correlation with severity of tinnitus [23]. This finding is in line with the model that the severity of tinnitus as elucidated by the questionnaires as well as associated emotional distress is less related to the causes of the condition and more to the cognitive factors of perception and reaction to tinnitus [20, 24]. When looking at the reverse effects of tinnitus on anxiety and depression, Gomaa et al. found that the duration of tinnitus had positive correlation on both severity of depression and severity of anxiety, but the severity of tinnitus did not affect severity of anxiety and depression [25]. In contrast to most literature, Ooms et al. found no correlation between depression and tinnitus and proposed that high correlations between the THI and BDI scores were due to significant content overlap [26]. The THI was validated against the BDI in its original development, and Langguth et al. further countered Ooms's argument with a study using quality of life measurements that indicated high THI scores reflecting a significant impairment in quality of life, independent of potential overlap in single items between the THI and BDI [27].

Our results showed that there were no differences in the mean PTAs among the five groups or in between ears in all the groups. The average hearing loss was around 38 dB HL. The lack of association between the PTA results and anxiety or depression suggests that these variables are independent in our veteran population. This finding was consistent with the study of Gomaa et al., demonstrating that hearing loss is not the dominant cause of anxiety/depression [25].

### 5.2. Mechanisms Associated with Anxiety/Depression and Tinnitus

Subjective tinnitus has been assumed to be caused by or associated with damage to the auditory system, both peripherally and centrally [28]. Early theories of tinnitus neural mechanisms suggested a peripheral generation model, where the origin of the phantom sound resides in the inner ear. The idea was based on the fact that cochlear damage from traumatizing sounds and ototoxic agents induced both hearing loss and tinnitus [29]. However, this idea was countered when bilateral auditory nerve sectioning did not always eliminate tinnitus [30]. Current measurements with magnetoencephalography (MEG) show increased spontaneous firing rate (SFR) of neurons in several auditory structures including the dorsal and ventral cochlear nucleus, the inferior colliculus, and auditory cortices, but no signals were seen in peripheral nerve fibers [28]. This evidence points to a central generation model, where all forms of tinnitus, even those triggered by cochlear damage, have origin in the central auditory system (CAS). The connection between peripheral damage and central auditory changes is explained as an alteration in the normal balance between excitatory and inhibitory nerve transmission brought about by loss of inhibition, which leads to increased firing rate [28, 31]. Auditory regions of the cerebral cortex have also been studied in relation to the Tonotopic Reorganization Model of tinnitus. The auditory cortex is organized tonotopically with specific anatomical sites relating to specific frequencies. According to Salvi et al., a lack of afferent signals from a specific region of the cochlea due to hearing loss leads to a rapid reduction in activity within the corresponding section of the auditory cortex, and neural plasticity allows new connections to adjacent cortical fields [32]. As a consequence of this reorganization, a disproportionately large number of neurons become sensitive to lower or high frequencies bordering on the area of hearing reduction. The spontaneous activity seen in this field may be perceived as tinnitus noise.

Functional MRI have shown increased signals in the middle and superior frontal gyri, the cingulate gyrus, the precuneus, and the parietal cortices in tinnitus patients, verified by EEG and MEG studies. The nonauditory structures are evidenced to play the greatest role in understanding the reaction to tinnitus, the subjective reporting of tinnitus severity, persistence of tinnitus perception, and physiological relationship to comorbid psychological factors such as anxiety and depression. Functional imaging studies show that the subgenual anterior cingulate cortex (sgACC) plays an important role in both coping styles [33] and depression [34]. The most recent study by Vanneste et al. showed that tinnitus patients using a maladaptive coping style show increased scores on the BDI and THI and experienced louder sounds and more distress in comparison to tinnitus patients using adaptive coping styles [35]. The sgACC and ventromedial prefrontal cortex are considered central dysfunction nodes in depression. The dorsolateral prefrontal cortex has been found to play an important role in anxiety [36]. These evidences further suggest why tinnitus can be associated with major depression, anxiety, and other psychosomatic and/or psychological disturbances.

### 5.3. Treatment of Tinnitus in Patients with Anxiety/Depression

Standard care of subjective tinnitus involves education/counseling, sound therapy (either hearing aids or sound generators), and intervention to reduce the distress (relaxation therapy or cognitive behavioral therapy (CBT) or both) [37]. The approach to using CBT for tinnitus patients follows the theory that relaxation and cognitive restructuring of thoughts may promote improved and habituated responses to the phantom noise, thus decreasing the distress level and perceived severity of tinnitus. The Cochrane Collaboration in 2007 reviewed 6 trials of CBT for tinnitus with 285 participants. Although the data analysis did not demonstrate any significant effect in the subjective loudness of tinnitus or comorbid depression, there was a significant improvement in quality of life, as assessed by the THI. This outcome further supports the idea that anxiety and depression are comorbidities that affect the perception and response to tinnitus [38].

At present no specific therapy for tinnitus is acknowledged to be satisfactory for all patients. Due to the high comorbidity of tinnitus and psychiatric illnesses [39], a wide range of pharmacological agents, including anticonvulsants, benzodiazepines, tricyclic antidepressants (including amitriptyline, imipramine, and nortriptyline), and selective serotonin reuptake inhibitors (SSRIs), have been used. However, there is debate about whether psychoactive drugs act on the central auditory system and reduce tinnitus directly, whether they act by treating concomitant psychological illnesses, or whether they have a simultaneous effect of both the psychological disturbance and tinnitus [40]. The Cochrane Study in 2012 evaluated six trials involving 610 patients with the object of assessing the effectiveness of antidepressants in the treatment of tinnitus and whether any benefit is due to a direct tinnitus effect or a secondary effect due to treatment of concomitant depressive states. They concluded that all trials assessing tricyclic antidepressants showed slight improvement in tinnitus, but the effects may have been attributed to methodological bias. The SSRI drug trial showed possible benefit for the subgroup that received higher doses of SSRIs, and the observation merits further investigation. The trial investigating trazodone showed an improvement in tinnitus intensity and quality of life, but it did not reach statistical significance. Overall, the Cochrane Collaboration concluded that further research is required because only one of the studies they reviewed met the high quality standard [41].

In conclusion, this study verified that strong correlations exist between the degree of tinnitus and anxiety and depression in a veteran population. Although tinnitus and anxiety/depression involve distinct perceptual events, they may share many mechanisms of the central nerve system, particularly those comprising the auditory cortical pathways and limbic system. A multidisciplinary team comprising psychiatrists and audiologists is needed to evaluate and manage the tinnitus patients with psychiatric comorbidity. There are still numerous uncertainties relating to tinnitus assessment, diagnosis, and treatment. We will further determine mechanisms of tinnitus, including whether particular neuronal dysfunction that drives tinnitus may also lower the threshold of susceptibility for anxiety/depression or vice versa.

## Figures and Tables

**Table 1:** The median age, standard deviations, and age ranges of veterans with tinnitus with/without anxiety and depression (*n* = 91).

Age parameters	Anxiety	No anxiety	Depression	No depression	Both	Neither
Median	62.5	61	64	65	65	64
SD	10.04	11.40	10.18	11.76	10.18	9.99
Range	31–73	51–80	31–79	31–84	39–79	51–84

**(a):** 

	Pearson correlation	Significance (2-tailed)
Total THI score *∗* anxiety	0.401	0.000
Functional THI score *∗* anxiety	0.474	0.000
Emotional THI score *∗* anxiety	0.473	0.000
Catastrophic THI score *∗* anxiety	0.279	0.006

**(b):** 

	Pearson correlation	Significance (2-tailed)
Total THI score *∗* depression	0.205	0.044
Functional THI score *∗* depression	0.429	0.000
Emotional THI score *∗* depression	0.331	0.001
Catastrophic THI score *∗* depression	0.271	0.007

**Table 3:** Tinnitus characteristics and duration.

Tinnitus character (Total)	Anxiety Total (%)	No anxiety Total (%)	Depression Total (%)	No depression Total (%)	Both Total (%)	Neither Total (%)
Intermittent (35)	28 (80)	4 (11.4)	18 (51.4)	14 (40)	18 (51.4)	4 (11.4)
Persistent (55)	37 (67.3)	10 (18.2)	33 (60)	16 (29.1)	30 (54.5)	9 (16.4)
Bilateral (84)	60 (71.4)	15 (17.9)	49 (58.3)	28 (33.3)	46 (54.8)	14 (16.7)
Unilateral (7)	6 (85.7)	1 (14.3)	4 (57.1)	3 (42.9)	3 (42.9)	0 (0)
More than 10 yrs. (45)	31 (15.6)	7 (15.6)	22 (48.9)	17 (37.8)	21 (46.7)	7 (15.6)
Less than 10 yrs. (21)	13 (19)	4 (19)	10 (47.6)	9 (42.9)	8 (38.1)	4 (19)
